# Supplementary material for: corseq: fast and efficient identification of favoured codons from next generation sequencing reads
Source: PeerJ. 2018 Jul 4;6:e5099. doi: 10.7717/peerj.5099 (PMC6035725; doi:10.7717/peerj.5099)
Supplement: File S1 — The file provides information about the proposed software usage. [file peerj-06-5099-s001.docx]

**corseq**

**Installation**

The software is implemented in Python 2.7 and does not necessitate any installation step. However the library Biopyhton should be installed in order for the software to work. Biopython installation is straightforward for Unix systems:

sudo easy_install -f http://biopython.org/DIST/ biopython

and for Mac OSX:

fink install biopython-pyXX

with XX representing the used python version. Please refer to the Biopython webpage for installation instruction under the Windows operative system

The file corseq.py and the folder “trainingSets” (this name must not be changed) can be moved into any desired directory or the original path included when running the program

**Usage**

corseq can be ran by using the following flags:

-i filename File name of the fastq RNAseq data

-ts Training set File name of the selected training set

-o output folder name Output folder name (default name: outputFolder)

-kp Protein kmer size Protein kmer from training set (default: 11)

-k Reads kmer size Sequence kmer for expression calculation (default: 39)

-n Number of bases Number of random bases (default 20000000)

-h, --help show this help message and exit

-kc kmer frequency cutoff Higher and lower nucleotide kmer percentile to define high and low kmer frequency

The first two arguments are compulsory and refer to the RNAseq fastq formatted file to be analysed and the protein training file necessary to populate the “Peptide set” respectively. The training set is just a multifasta formatted protein file containing sequences of species that are phylogenetically close to that under investigation. The software comes with the trainingSets folder already populated with four training sets (plants, fungi, bacteria and metazoan) that are relative to the four main phylogenetic groups and that were obtained by the Uniprot database. However, the user can decide to use a more specific protein set (i.e. proteins belonging to organisms that are phylogenetically very close to that under investigation) by adding the corresponding protein fasta file in the trainingSets folder. All the possible amino acid kmers are extracted from the provided proteins and used to create the “Peptide set”. The default size of this kmer is set to 11 but can be tuned by the user with the flag –kp. In principle highest values correspond to a higher specificity and lower sensitivity. In our benchmarks a value of 11 proved to work satisfyingly.

The flag –o is used to indicate the name of the output folder. If not output folder is indicated, all the results will be created in a directory called “outputFolder”. If the output folder already exists corseq will return a warning message and exit the main prompt.

The Peptides sets is used to individuate the correct frame and orientation of the provided RNAseq reads with this step producing several thousands of psedo-ORF (see the original paper for detail). Such pseudo-ORFs are subjected to a nucleotide kmer analysis in order to investigate the most and less recurrent kmers that are likely to derive from highly and lowly expressed genes. Again higher kmer sizes are associate with higher specificity and lower sensitivity. In our test a kmer size of 39 proved to produce reliable results.

Finally, in order to reduce the running time the user can select a number of bases to run the corseq analysis. The software infer the corresponding number of reads and randomly extract them from the original RNAseq file. Our benchmark underlined an increase in performance with the selected number of bases and the default value 20000000 allowing the maximum reliability while keeping the running time low (<3 hours for fastq file size >5.8Gb)

During operation some warning may appear related to the Biopython module Seq.translate(). This is something does not influence the analysis and can be ignored.

**Output files**

corseq will generate the following files in the output folder (whose name is define by the flag –o)

*codonTable.txt*: the list of the favoured codons

*codonTable_statistics.txt*: a table reporting, for each codon, the calculated odds ratio, the significance and the number of occurrence in highly and lowly represented kmers.

*kmerDist.txt*: the number of occurrences for each nucleotide kmers

*logFile.txt*: the file with the run log

*highRepresentativeKmer.fasta*: a multifasta file reporting the highly represented nucleotide kmers

*lowRepresentativeKmer.fasta*: a multifasta file reporting the lowly represented nucleotide kmers

*reads_temp.fasta*: a multifasta file reporting the reads that have been randomly extracted from the initial RNAseq fastq formatted file

*readsInFrame.fasta*: a multifasta file reporting the pseuso-ORF

**Examples**:

1. The following command is used to compute the favoured codons for an plant organism by computing its RNAseq file that is named myRNA.fastq. All the remaining parameters are left in their default values

python corseq –i myRNA.fastq –ts plants

1. The following command will use the same RNAseq file as described in example (a) but in this case the user prefer to use the Oryza sativa proteins as the training dataset. If we assume that the protein multifasta file is named osativa_prot.fasta the following command will do the job (the file osativa_prot.fasta must be copied into the “trainingSets” folder)

python corseq –i myRNA.fastq –ts osativa_prot.fasta

1. The following command will randomly extract 4,000,000 bp from the input RNAseq file (instead of the default value 20,000,000) and will use a reads kmer size of 24 (instead of the default value of 39)

python corseq –i myRNA.fastq –ts plants –n 4000000 –k 24
