# Supplementary material for: corseq: fast and efficient identification of favoured codons from next generation sequencing reads
Source: PeerJ. 2018 Jul 4;6:e5099. doi: 10.7717/peerj.5099 (PMC6035725; doi:10.7717/peerj.5099)
Supplement: File S2 — In this file, a study is reported dealing with the effect the nucleotide kmer frequency percentage cutoff on the output of the corseq pipeline. [file peerj-06-5099-s002.docx]

**The effect of the kmer frequency cutoff**

As reported in the main manuscript, the identified pseudo-ORFs are dissected in their kmers whose total number of occurrences is computed. Kmers are then sorted by their number of occurrences and the 2% most frequent and 2% less frequent kmers are used as a proxy to infer motifs that are representative of highly and lowly expressed transcripts respectively. While 2% represent the default value for the corseq pipeline, we wondered whether such a parameter may play a role in the obtained results. For this reason we repeated our analyses by setting this parameter to the following values: 1%, 2%, 5%, 10% and 15%. All the remaining parameters were left in their default values (e.g. number of bases = 20Mb, kmer size = 39). The analysis was performed for the species *A. thaliana*, *S. cerevisiae*, *M. musculus*, *E. coli* and *D. melanogaster*. For each species we reported the number of found favoured codons into Venn diagrams with intersection reporting the number of favoured codons that proved to be concordant among the used percentage cutoffs. As shown in Figure S2_1, the obtained results proved to be consistent in the range of the used cutoffs.


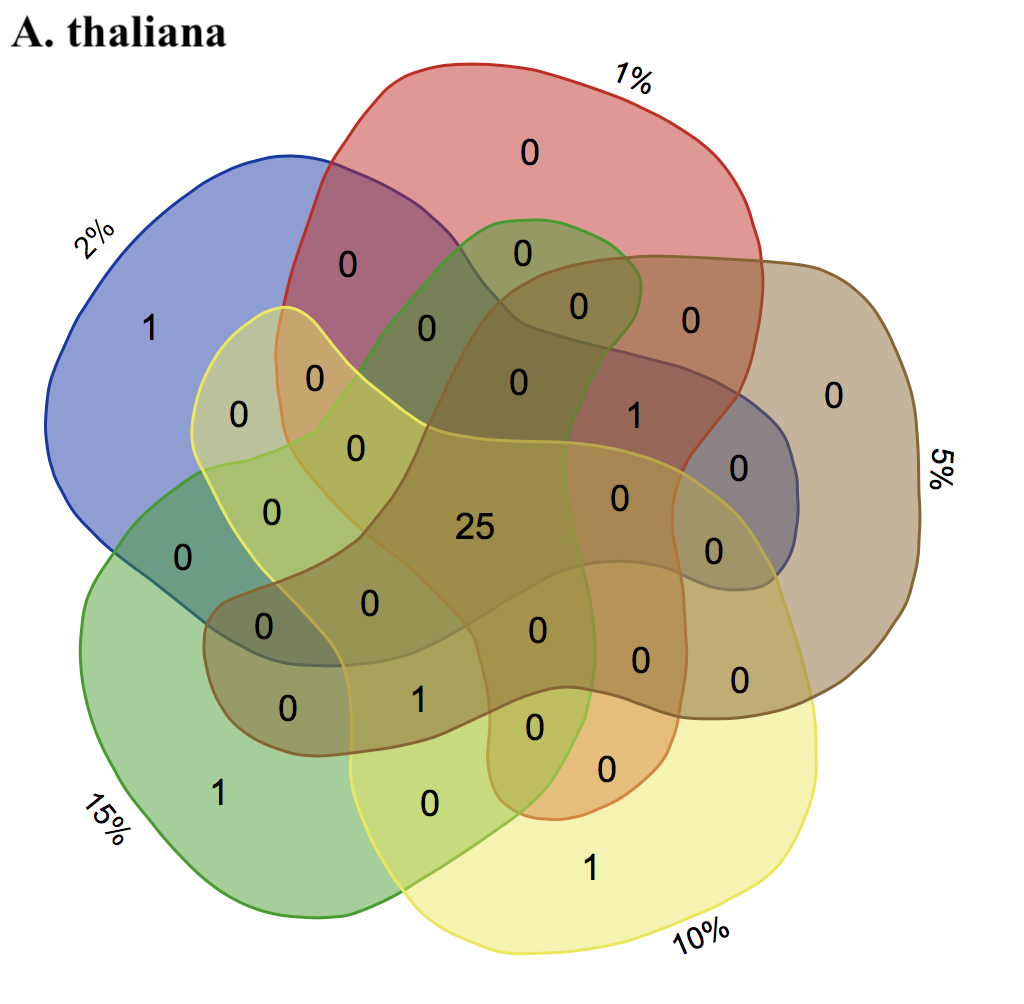

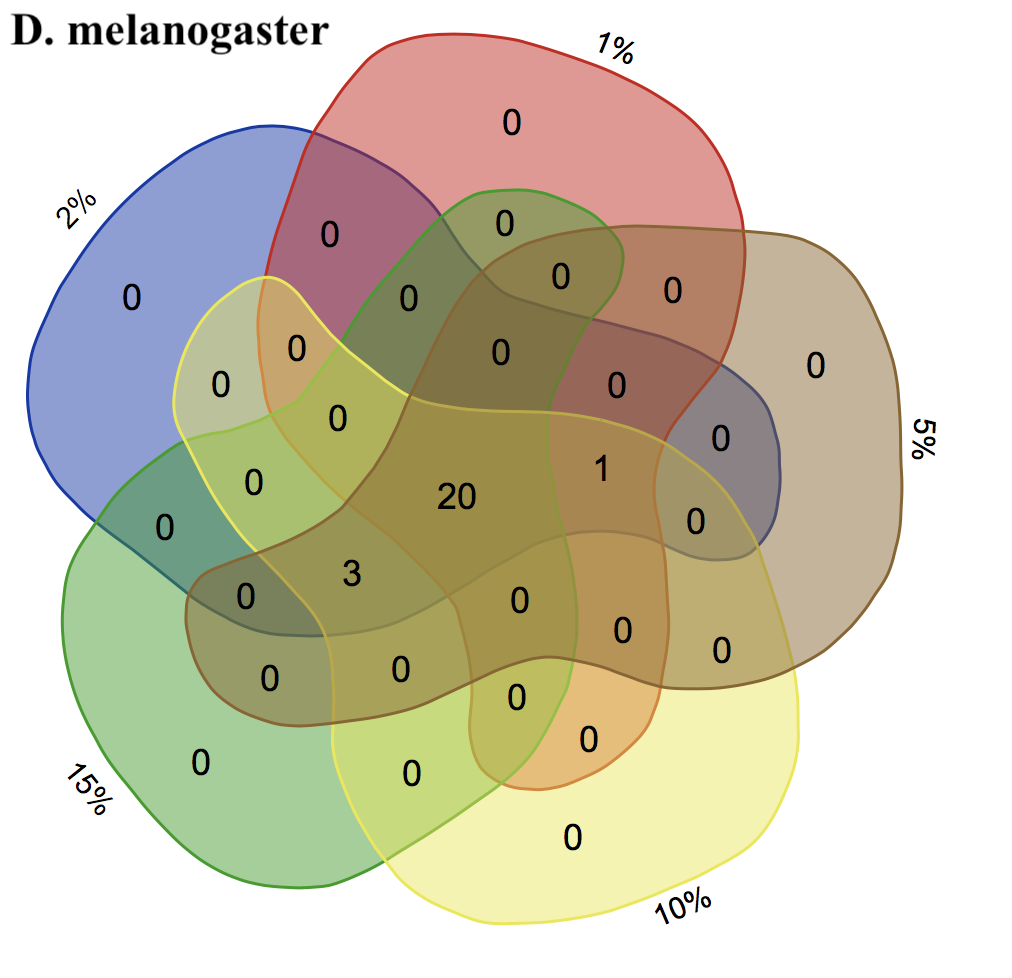

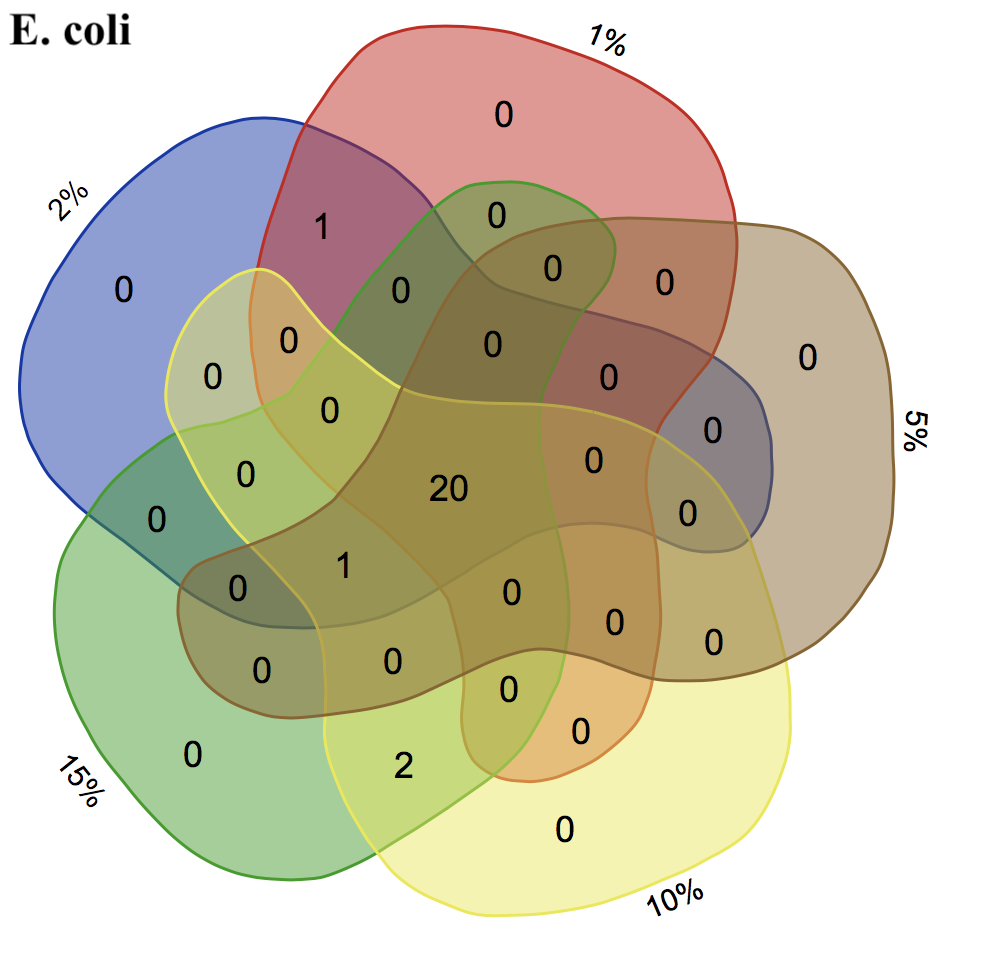


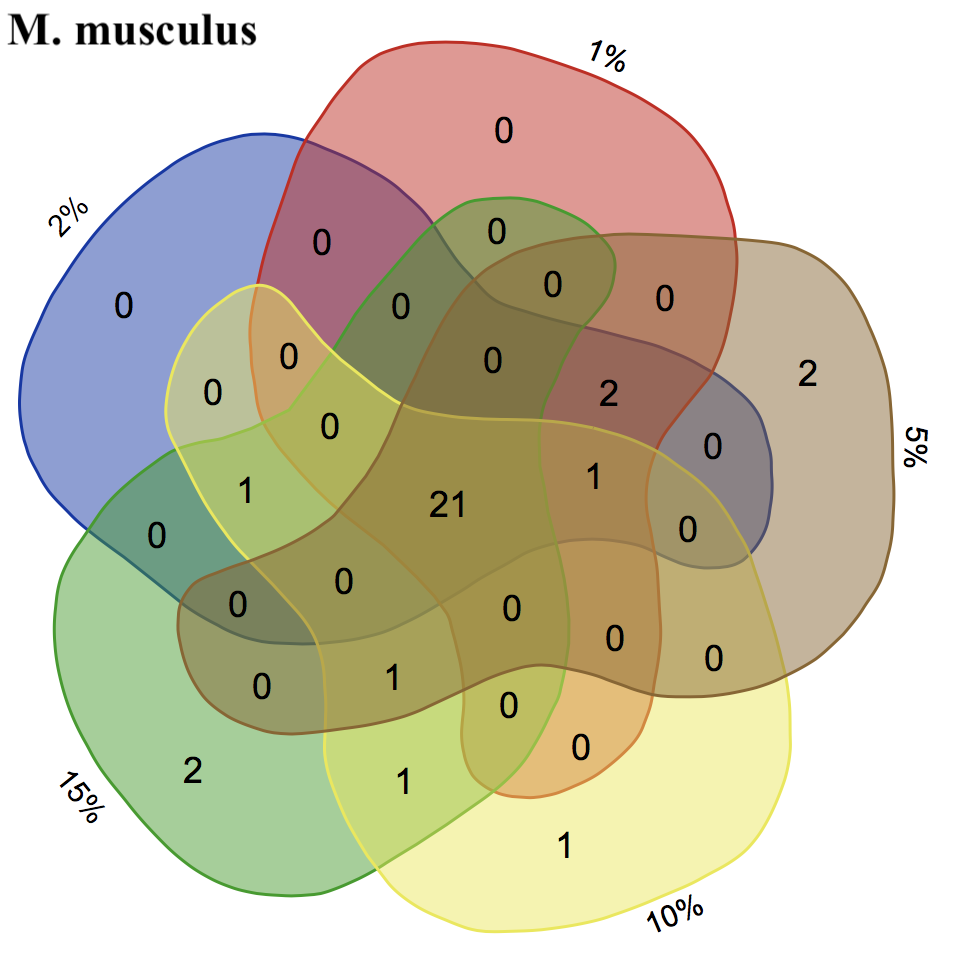

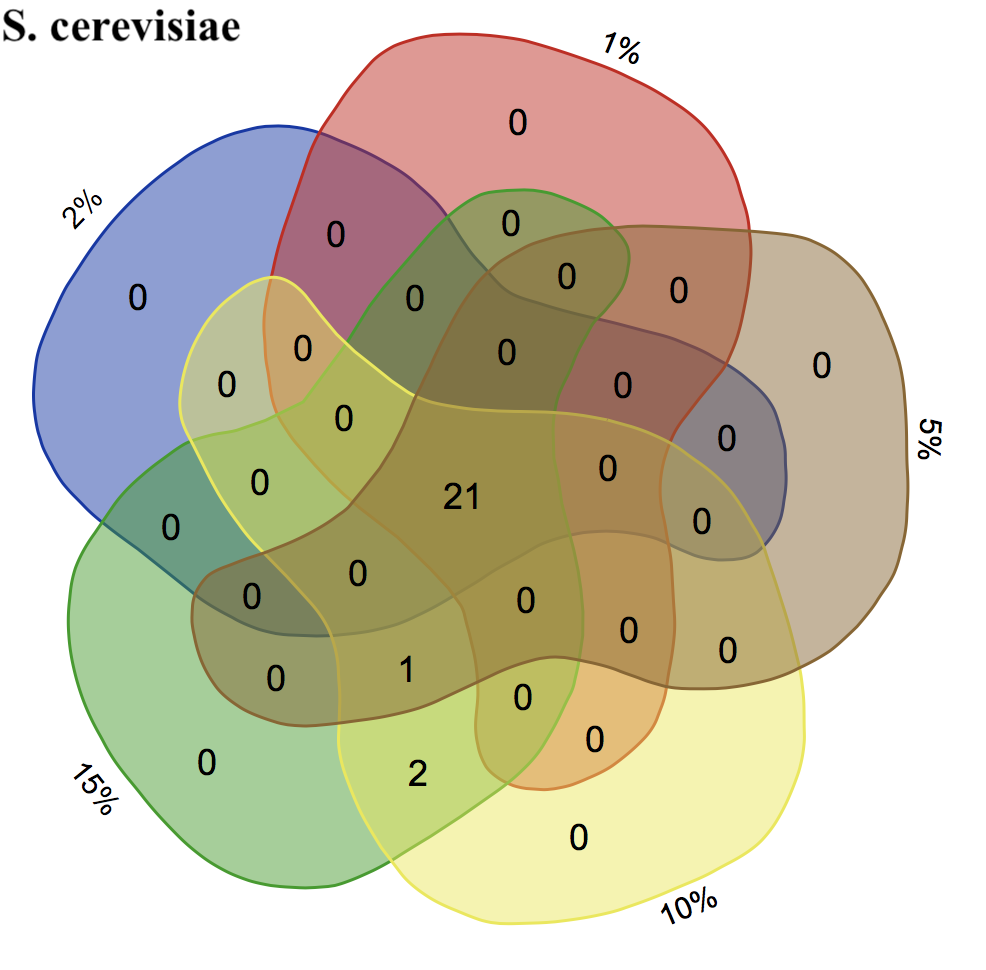


**Figure S2_1**: Venn diagrams reporting the number of favoured codons found with the corseq pipeline while changing the kmer frequency percentage cutoff. Each set represent the number of favoured codons found with a specific cutoff and overlapping regions report the number of concordant codons.

As expected, increasing the percentage cutoff reduces the difference between the number of occurrences of high and low frequent kmers (Figure S2_2). Since such frequency is considered as a proxy of the expression profile, a high divergence in the kmer occurrence between these two datasets should better capture the differences in the codon usage. For this reason a lower percentage should be advisable.

**Figure S2_2**: kmer frequency divergence difference between high and low frequent kmer occurrence at different percentage cutoffs for 5 species. Values on the y axis is calculated as the difference between the minimum occurrences of highly frequent kmers and the maximum occurrences of the low frequency kmers.

Notably, decreasing the percentage cutoff imply a lower number of analysed kmers (Figure S2_3). Therefore, attention should to be paid in avoiding too low values that may diminish the power of the statistical analysis. The number of the analysed kmers is reported in the logFile.txt generated by corseq at the end of the computation.

**Figure S2_3**: Number of analysed kmers (only high frequency kmers are shown) as a function of the percentage cutoff
