# Supplementary material for: corseq: fast and efficient identification of favoured codons from next generation sequencing reads
Source: PeerJ. 2018 Jul 4;6:e5099. doi: 10.7717/peerj.5099 (PMC6035725; doi:10.7717/peerj.5099)
Supplement: File S3 — Statistics are reported related to the odds ratio of each codon for each species with both the corseq pipeline and the conventional expression method. [file peerj-06-5099-s003.docx]

Hereafter the odds ratio and the significance values are reported for corseq and CEM

*Arabidopsis thaliana*

|  |  | **CEM** | | **corseq** | |
| --- | --- | --- | --- | --- | --- |
| **AA** | **Codon** | **Odds ratio** | **Significance (Z^2^-score)** | **Odds ratio** | **Significance (Z^2^-score)** |
| A | GCA | 0.63 | 620.57 | 0.67 | 1013.26 |
| A | GCC | 1.15 | 49.56 | 1.36 | 551.01 |
| A | GCG | 0.96 | 3.87 | 0.72 | 363.88 |
| A | GCT | 1.32 | 321.34 | 1.28 | 583.68 |
| C | TGC | 1.01 | 0.06 | 1.31 | 130.95 |
| C | TGT | 0.99 | 0.06 | 0.76 | 130.95 |
| E | GAA | 0.70 | 485.97 | 0.57 | 2520.10 |
| E | GAG | 1.42 | 485.97 | 1.74 | 2520.10 |
| D | GAC | 1.15 | 63.56 | 1.66 | 1804.65 |
| D | GAT | 0.87 | 63.56 | 0.60 | 1804.65 |
| G | GGA | 0.93 | 19.52 | 0.98 | 3.07 |
| G | GGC | 0.85 | 50.13 | 0.88 | 67.86 |
| G | GGG | 0.74 | 180.20 | 0.56 | 1202.11 |
| G | GGT | 1.36 | 378.64 | 1.41 | 1171.06 |
| F | TTC | 1.45 | 374.45 | 1.85 | 1913.46 |
| F | TTT | 0.69 | 374.45 | 0.54 | 1913.46 |
| I | ATA | 0.44 | 1322.51 | 0.32 | 3471.84 |
| I | ATC | 1.74 | 1006.62 | 2.08 | 3963.11 |
| I | ATT | 0.98 | 0.90 | 0.89 | 98.87 |
| H | CAC | 1.51 | 231.62 | 2.08 | 1719.43 |
| H | CAT | 0.66 | 231.62 | 0.48 | 1719.43 |
| K | AAA | 0.65 | 764.83 | 0.46 | 5583.86 |
| K | AAG | 1.54 | 764.83 | 2.19 | 5583.86 |
| L | CTA | 0.73 | 198.41 | 0.63 | 579.55 |
| L | CTC | 1.35 | 324.85 | 1.47 | 1049.51 |
| L | CTG | 0.85 | 46.44 | 0.80 | 169.34 |
| L | CTT | 1.19 | 130.31 | 1.32 | 695.78 |
| L | TTA | 0.66 | 391.04 | 0.41 | 1960.22 |
| L | TTG | 1.03 | 3.56 | 1.08 | 39.67 |
| N | AAC | 1.50 | 425.33 | 2.37 | 3397.14 |
| N | AAT | 0.67 | 425.33 | 0.42 | 3397.14 |
| Q | CAA | 0.65 | 377.61 | 0.67 | 638.37 |
| Q | CAG | 1.54 | 377.61 | 1.49 | 638.37 |
| P | CCA | 0.87 | 55.79 | 1.11 | 61.82 |
| P | CCC | 1.00 | 0.02 | 1.12 | 31.89 |
| P | CCG | 1.17 | 46.50 | 0.83 | 101.65 |
| P | CCT | 1.04 | 4.09 | 0.96 | 12.23 |
| S | AGC | 0.98 | 0.67 | 1.20 | 140.32 |
| S | AGT | 0.85 | 64.22 | 0.87 | 77.11 |
| S | TCA | 0.81 | 141.36 | 0.81 | 207.08 |
| S | TCC | 1.16 | 56.08 | 1.32 | 337.28 |
| S | TCG | 1.17 | 51.87 | 0.82 | 100.26 |
| S | TCT | 1.11 | 46.28 | 1.06 | 21.50 |
| R | AGA | 0.83 | 97.70 | 0.82 | 223.88 |
| R | AGG | 1.20 | 68.10 | 1.46 | 770.25 |
| R | CGA | 0.68 | 160.43 | 0.43 | 1013.92 |
| R | CGC | 0.95 | 1.98 | 1.02 | 0.57 |
| R | CGG | 0.71 | 99.54 | 0.32 | 1200.82 |
| R | CGT | 1.61 | 463.14 | 1.77 | 1692.29 |
| T | ACA | 0.62 | 584.78 | 0.62 | 1172.50 |
| T | ACC | 1.45 | 343.19 | 1.78 | 2072.65 |
| T | ACG | 0.91 | 14.25 | 0.68 | 396.26 |
| T | ACT | 1.19 | 95.58 | 1.12 | 92.64 |
| V | GTA | 0.56 | 589.91 | 0.44 | 1758.37 |
| V | GTC | 1.20 | 98.93 | 1.46 | 1015.83 |
| V | GTG | 1.01 | 0.20 | 1.12 | 95.35 |
| V | GTT | 1.14 | 70.59 | 0.96 | 15.25 |
| Y | TAC | 1.76 | 570.75 | 2.30 | 2734.69 |
| Y | TAT | 0.57 | 570.75 | 0.43 | 2734.69 |

*Oryza sativa*

|  |  | **CEM** | | **corseq** | |
| --- | --- | --- | --- | --- | --- |
| **AA** | **Codon** | **Odds ratio** | **Significance (Z^2^-score)** | **Odds ratio** | **Significance (Z^2^-score)** |
| A | GCA | 0.44 | 1822.86 | 0.57 | 889.36 |
| A | GCC | 1.50 | 862.21 | 1.62 | 1272.81 |
| A | GCG | 1.41 | 565.06 | 0.86 | 79.30 |
| A | GCT | 0.68 | 499.23 | 0.98 | 1.23 |
| C | TGC | 2.48 | 604.35 | 2.10 | 456.38 |
| C | TGT | 0.40 | 604.35 | 0.48 | 456.38 |
| E | GAA | 0.37 | 2301.59 | 0.47 | 1618.40 |
| E | GAG | 2.67 | 2301.59 | 2.13 | 1618.40 |
| D | GAC | 2.15 | 1604.89 | 1.70 | 1025.71 |
| D | GAT | 0.47 | 1604.89 | 0.59 | 1025.71 |
| G | GGA | 0.64 | 487.48 | 0.72 | 309.09 |
| G | GGC | 1.58 | 919.07 | 1.58 | 1109.52 |
| G | GGG | 0.97 | 2.66 | 0.67 | 489.20 |
| G | GGT | 0.75 | 219.97 | 1.03 | 3.07 |
| F | TTC | 2.42 | 1244.93 | 1.97 | 926.34 |
| F | TTT | 0.41 | 1244.92 | 0.51 | 926.34 |
| I | ATA | 0.36 | 1121.10 | 0.30 | 1509.26 |
| I | ATC | 2.83 | 2448.52 | 2.55 | 2838.56 |
| I | ATT | 0.56 | 636.04 | 0.64 | 547.70 |
| H | CAC | 2.36 | 843.78 | 1.71 | 374.87 |
| H | CAT | 0.42 | 843.78 | 0.58 | 374.87 |
| K | AAA | 0.31 | 2630.36 | 0.29 | 2895.97 |
| K | AAG | 3.27 | 2630.36 | 3.39 | 2895.97 |
| L | CTA | 0.42 | 676.78 | 0.40 | 646.28 |
| L | CTC | 1.94 | 2004.93 | 1.76 | 1696.07 |
| L | CTG | 1.40 | 447.17 | 1.35 | 406.52 |
| L | CTT | 0.71 | 293.09 | 0.92 | 27.75 |
| L | TTA | 0.31 | 918.86 | 0.19 | 1000.05 |
| L | TTG | 0.54 | 753.30 | 0.53 | 781.56 |
| N | AAC | 2.86 | 1742.68 | 2.70 | 1961.69 |
| N | AAT | 0.35 | 1742.68 | 0.37 | 1961.69 |
| Q | CAA | 0.38 | 1266.16 | 0.43 | 1032.85 |
| Q | CAG | 2.64 | 1266.16 | 2.32 | 1032.85 |
| P | CCA | 0.50 | 945.56 | 0.73 | 222.93 |
| P | CCC | 1.60 | 503.33 | 1.38 | 258.54 |
| P | CCG | 1.56 | 550.75 | 1.17 | 59.63 |
| P | CCT | 0.68 | 310.47 | 0.86 | 50.14 |
| S | AGC | 1.23 | 123.09 | 1.43 | 418.40 |
| S | AGT | 0.49 | 627.84 | 0.64 | 251.98 |
| S | TCA | 0.47 | 957.84 | 0.56 | 583.90 |
| S | TCC | 1.70 | 905.05 | 1.46 | 495.96 |
| S | TCG | 1.73 | 717.92 | 1.05 | 5.53 |
| S | TCT | 0.61 | 467.00 | 0.81 | 92.06 |
| R | AGA | 0.38 | 1249.04 | 0.42 | 801.60 |
| R | AGG | 1.02 | 0.84 | 1.49 | 488.52 |
| R | CGA | 0.56 | 251.04 | 0.39 | 412.11 |
| R | CGC | 1.89 | 1148.43 | 1.68 | 806.59 |
| R | CGG | 1.13 | 33.07 | 0.56 | 410.15 |
| R | CGT | 0.91 | 10.44 | 1.17 | 40.99 |
| T | ACA | 0.41 | 1211.22 | 0.46 | 1109.93 |
| T | ACC | 2.03 | 1260.41 | 2.29 | 2512.26 |
| T | ACG | 1.40 | 219.81 | 0.85 | 47.35 |
| T | ACT | 0.59 | 431.63 | 0.73 | 207.27 |
| V | GTA | 0.39 | 848.09 | 0.35 | 861.87 |
| V | GTC | 1.62 | 803.82 | 1.39 | 472.54 |
| V | GTG | 1.25 | 183.23 | 1.29 | 291.37 |
| V | GTT | 0.57 | 759.52 | 0.70 | 409.00 |
| Y | TAC | 2.56 | 1059.34 | 2.48 | 1188.29 |
| Y | TAT | 0.39 | 1059.34 | 0.40 | 1188.29 |

*Echerichia coli*

|  |  | **CEM** | | **corseq** | |
| --- | --- | --- | --- | --- | --- |
| **AA** | **Codon** | **Odds ratio** | **Significance (Z^2^-score)** | **Odds ratio** | **Significance (Z^2^-score)** |
| A | GCA | 1.33 | 29.46 | 1.36 | 266.21 |
| A | GCC | 0.44 | 203.31 | 0.48 | 1069.50 |
| A | GCG | 0.62 | 88.73 | 0.46 | 1547.45 |
| A | GCT | 2.79 | 393.18 | 2.97 | 4481.75 |
| C | TGC | 1.32 | 3.23 | 1.38 | 27.36 |
| C | TGT | 0.76 | 3.23 | 0.73 | 27.36 |
| E | GAA | 1.37 | 25.50 | 1.48 | 271.17 |
| E | GAG | 0.73 | 25.50 | 0.67 | 271.17 |
| D | GAC | 2.20 | 163.71 | 2.13 | 1143.06 |
| D | GAT | 0.45 | 163.71 | 0.47 | 1143.06 |
| G | GGA | 0.27 | 141.71 | 0.22 | 736.24 |
| G | GGC | 1.07 | 1.91 | 0.93 | 16.93 |
| G | GGG | 0.23 | 217.97 | 0.15 | 1505.53 |
| G | GGT | 2.26 | 252.93 | 2.39 | 2368.18 |
| F | TTC | 2.89 | 186.50 | 2.96 | 1211.30 |
| F | TTT | 0.35 | 186.50 | 0.34 | 1211.30 |
| I | ATA | 0.26 | 70.34 | 0.17 | 348.75 |
| I | ATC | 2.57 | 271.26 | 3.02 | 2297.48 |
| I | ATT | 0.49 | 155.46 | 0.39 | 1553.12 |
| H | CAC | 2.47 | 81.36 | 2.54 | 522.09 |
| H | CAT | 0.40 | 81.36 | 0.39 | 522.09 |
| K | AAA | 0.87 | 3.87 | 0.94 | 7.09 |
| K | AAG | 1.15 | 3.87 | 1.07 | 7.09 |
| L | CTA | 0.38 | 31.64 | 0.24 | 248.12 |
| L | CTC | 0.63 | 28.33 | 0.67 | 135.55 |
| L | CTG | 2.90 | 465.81 | 2.65 | 2303.22 |
| L | CTT | 0.68 | 19.24 | 0.63 | 163.78 |
| L | TTA | 0.33 | 159.17 | 0.29 | 674.81 |
| L | TTG | 0.50 | 68.32 | 0.54 | 301.45 |
| N | AAC | 3.23 | 217.06 | 2.98 | 1155.44 |
| N | AAT | 0.31 | 217.06 | 0.34 | 1155.44 |
| Q | CAA | 0.55 | 61.35 | 0.56 | 316.22 |
| Q | CAG | 1.83 | 61.35 | 1.80 | 316.22 |
| P | CCA | 0.91 | 1.03 | 0.78 | 41.30 |
| P | CCC | 0.35 | 53.40 | 0.28 | 365.60 |
| P | CCG | 1.72 | 53.50 | 1.81 | 414.49 |
| P | CCT | 0.77 | 6.11 | 0.86 | 14.51 |
| S | AGC | 0.96 | 0.23 | 0.78 | 71.40 |
| S | AGT | 0.35 | 90.54 | 0.36 | 361.52 |
| S | TCA | 0.51 | 38.01 | 0.46 | 207.42 |
| S | TCC | 1.66 | 43.93 | 1.75 | 388.27 |
| S | TCG | 0.38 | 76.41 | 0.29 | 533.06 |
| S | TCT | 2.98 | 232.94 | 2.91 | 1757.56 |
| R | AGA | 0.28 | 40.20 | 0.20 | 147.33 |
| R | AGG | 0.50 | 6.68 | 0.27 | 90.72 |
| R | CGA | 0.23 | 71.07 | 0.18 | 316.12 |
| R | CGC | 0.68 | 38.18 | 0.67 | 275.88 |
| R | CGG | 0.24 | 107.21 | 0.19 | 537.20 |
| R | CGT | 3.13 | 355.41 | 2.80 | 2028.86 |
| T | ACA | 0.42 | 66.00 | 0.33 | 413.92 |
| T | ACC | 1.21 | 10.26 | 1.22 | 77.34 |
| T | ACG | 0.38 | 137.50 | 0.33 | 976.17 |
| T | ACT | 2.73 | 207.27 | 2.55 | 1605.95 |
| V | GTA | 1.72 | 68.38 | 1.52 | 364.56 |
| V | GTC | 0.54 | 80.31 | 0.50 | 610.34 |
| V | GTG | 0.43 | 215.62 | 0.40 | 1617.65 |
| V | GTT | 2.31 | 244.01 | 2.43 | 2413.85 |
| Y | TAC | 2.74 | 122.47 | 2.21 | 557.19 |
| Y | TAT | 0.37 | 122.47 | 0.45 | 557.19 |

*Staphylococcus aureus*

|  |  | **CEM** | | **corseq** | |
| --- | --- | --- | --- | --- | --- |
| **AA** | **Codon** | **Odds ratio** | **Significance (Z^2^-score)** | **Odds ratio** | **Significance (Z^2^-score)** |
| A | GCA | 1.11 | 2.59 | 1.10 | 10.73 |
| A | GCC | 0.68 | 7.20 | 0.58 | 51.44 |
| A | GCG | 0.58 | 36.29 | 0.88 | 8.64 |
| A | GCT | 1.36 | 21.21 | 1.07 | 4.98 |
| C | TGC | 1.12 | 0.21 | 1.25 | 3.30 |
| C | TGT | 0.89 | 0.21 | 0.80 | 3.30 |
| E | GAA | 1.46 | 17.22 | 1.14 | 14.62 |
| E | GAG | 0.69 | 17.22 | 0.87 | 14.62 |
| D | GAC | 1.60 | 40.04 | 1.13 | 17.66 |
| D | GAT | 0.62 | 40.04 | 0.88 | 17.66 |
| G | GGA | 0.98 | 0.06 | 0.96 | 0.82 |
| G | GGC | 0.83 | 3.63 | 0.83 | 16.47 |
| G | GGG | 0.43 | 28.56 | 0.88 | 3.73 |
| G | GGT | 1.38 | 20.70 | 1.16 | 22.00 |
| F | TTC | 1.93 | 50.73 | 1.05 | 1.47 |
| F | TTT | 0.52 | 50.73 | 0.95 | 1.47 |
| I | ATA | 0.70 | 19.05 | 0.85 | 17.59 |
| I | ATC | 1.28 | 10.44 | 0.99 | 0.06 |
| I | ATT | 1.06 | 0.88 | 1.11 | 12.34 |
| H | CAC | 1.96 | 23.77 | 1.09 | 2.14 |
| H | CAT | 0.51 | 23.77 | 0.92 | 2.14 |
| K | AAA | 1.98 | 67.29 | 1.47 | 74.76 |
| K | AAG | 0.51 | 67.29 | 0.68 | 74.76 |
| L | CTA | 0.92 | 0.49 | 0.90 | 5.53 |
| L | CTC | 0.65 | 4.14 | 0.51 | 31.78 |
| L | CTG | 0.38 | 17.42 | 0.89 | 2.13 |
| L | CTT | 1.02 | 0.03 | 0.96 | 1.14 |
| L | TTA | 1.44 | 37.13 | 1.26 | 70.12 |
| L | TTG | 0.64 | 25.23 | 0.75 | 35.83 |
| N | AAC | 1.37 | 19.16 | 1.08 | 6.57 |
| N | AAT | 0.73 | 19.16 | 0.92 | 6.57 |
| Q | CAA | 1.40 | 5.44 | 1.20 | 8.90 |
| Q | CAG | 0.71 | 5.44 | 0.84 | 8.90 |
| P | CCA | 1.20 | 3.09 | 1.43 | 68.66 |
| P | CCC | 0.59 | 3.02 | 0.52 | 18.18 |
| P | CCG | 0.68 | 5.61 | 0.60 | 35.60 |
| P | CCT | 1.06 | 0.28 | 0.86 | 10.25 |
| S | AGC | 1.20 | 2.72 | 1.18 | 14.01 |
| S | AGT | 0.74 | 16.07 | 0.91 | 7.92 |
| S | TCA | 1.46 | 31.69 | 1.15 | 26.07 |
| S | TCC | 0.71 | 2.09 | 0.74 | 12.47 |
| S | TCG | 0.42 | 30.72 | 0.87 | 6.82 |
| S | TCT | 1.04 | 0.23 | 0.93 | 5.30 |
| R | AGA | 0.84 | 3.29 | 0.94 | 1.80 |
| R | AGG | 0.61 | 4.46 | 0.46 | 24.42 |
| R | CGA | 0.60 | 12.55 | 0.84 | 7.97 |
| R | CGC | 0.83 | 0.96 | 0.64 | 29.92 |
| R | CGG | 0.73 | 0.46 | 0.26 | 25.98 |
| R | CGT | 1.74 | 36.54 | 1.42 | 84.65 |
| T | ACA | 1.15 | 4.14 | 1.08 | 6.92 |
| T | ACC | 0.36 | 21.94 | 0.52 | 44.48 |
| T | ACG | 0.55 | 34.73 | 0.81 | 20.03 |
| T | ACT | 1.40 | 21.54 | 1.11 | 11.82 |
| V | GTA | 1.36 | 22.08 | 1.15 | 23.36 |
| V | GTC | 0.46 | 49.77 | 0.71 | 42.31 |
| V | GTG | 0.62 | 26.71 | 0.81 | 21.15 |
| V | GTT | 1.29 | 16.33 | 1.06 | 4.72 |
| Y | TAC | 1.51 | 16.90 | 0.99 | 0.08 |
| Y | TAT | 0.66 | 16.90 | 1.01 | 0.08 |

*Lactobacillus pentosus*

|  |  | **CEM** | | **corseq** | |
| --- | --- | --- | --- | --- | --- |
| **AA** | **Codon** | **Odds ratio** | **Significance (Z^2^-score)** | **Odds ratio** | **Significance (Z^2^-score)** |
| A | GCA | 1.15 | 4.39 | 0.95 | 3.94 |
| A | GCC | 0.83 | 9.80 | 1.11 | 15.75 |
| A | GCG | 0.30 | 251.08 | 0.51 | 418.70 |
| A | GCT | 2.76 | 284.64 | 1.49 | 289.64 |
| C | TGC | 1.45 | 1.23 | 1.08 | 0.34 |
| C | TGT | 0.69 | 1.23 | 0.93 | 0.34 |
| E | GAA | 4.80 | 163.64 | 3.99 | 644.22 |
| E | GAG | 0.21 | 163.64 | 0.25 | 644.22 |
| D | GAC | 1.30 | 14.41 | 1.02 | 0.51 |
| D | GAT | 0.77 | 14.41 | 0.98 | 0.51 |
| G | GGA | 0.55 | 26.35 | 0.57 | 150.91 |
| G | GGC | 0.68 | 30.59 | 0.90 | 14.76 |
| G | GGG | 0.45 | 74.16 | 0.85 | 17.95 |
| G | GGT | 2.75 | 239.53 | 1.44 | 212.17 |
| F | TTC | 2.34 | 88.06 | 1.33 | 54.07 |
| F | TTT | 0.43 | 88.06 | 0.75 | 54.07 |
| I | ATA | 0.28 | 13.75 | 0.10 | 77.06 |
| I | ATC | 1.66 | 54.92 | 0.82 | 50.38 |
| I | ATT | 0.65 | 39.46 | 1.31 | 90.58 |
| H | CAC | 1.15 | 1.36 | 1.13 | 6.39 |
| H | CAT | 0.87 | 1.36 | 0.89 | 6.39 |
| K | AAA | 0.48 | 106.70 | 0.41 | 992.52 |
| K | AAG | 2.07 | 106.70 | 2.46 | 992.52 |
| L | CTA | 0.42 | 46.54 | 0.77 | 17.81 |
| L | CTC | 0.48 | 48.61 | 0.41 | 308.67 |
| L | CTG | 0.26 | 172.43 | 0.37 | 428.69 |
| L | CTT | 2.28 | 73.34 | 0.92 | 4.66 |
| L | TTA | 2.09 | 153.21 | 1.98 | 750.58 |
| L | TTG | 1.13 | 3.41 | 1.20 | 46.94 |
| N | AAC | 2.96 | 167.54 | 1.50 | 118.43 |
| N | AAT | 0.34 | 167.54 | 0.67 | 118.43 |
| Q | CAA | 3.84 | 183.90 | 2.43 | 338.27 |
| Q | CAG | 0.26 | 183.90 | 0.41 | 338.27 |
| P | CCA | 1.73 | 34.81 | 1.47 | 115.67 |
| P | CCC | 0.33 | 43.54 | 0.58 | 40.08 |
| P | CCG | 0.22 | 139.77 | 0.29 | 459.31 |
| P | CCT | 3.98 | 153.05 | 1.64 | 172.40 |
| S | AGC | 0.72 | 11.29 | 0.89 | 6.43 |
| S | AGT | 0.56 | 46.73 | 0.97 | 0.50 |
| S | TCA | 2.68 | 160.46 | 1.60 | 221.43 |
| S | TCC | 1.16 | 1.59 | 0.96 | 0.73 |
| S | TCG | 0.27 | 101.79 | 0.39 | 257.64 |
| S | TCT | 1.97 | 38.52 | 1.06 | 1.72 |
| R | AGA | 0.56 | 3.35 | 0.07 | 66.50 |
| R | AGG | 0.03 | 14.36 | 0.01 | 59.43 |
| R | CGA | 0.22 | 69.51 | 0.61 | 33.58 |
| R | CGC | 0.64 | 19.30 | 0.61 | 116.37 |
| R | CGG | 0.80 | 5.97 | 1.79 | 256.01 |
| R | CGT | 3.40 | 194.05 | 1.13 | 12.95 |
| T | ACA | 0.88 | 1.48 | 0.56 | 160.84 |
| T | ACC | 0.66 | 28.14 | 0.92 | 5.55 |
| T | ACG | 0.65 | 39.73 | 0.78 | 59.99 |
| T | ACT | 2.66 | 184.55 | 1.83 | 437.65 |
| V | GTA | 1.53 | 15.85 | 0.58 | 142.58 |
| V | GTC | 0.47 | 125.98 | 0.65 | 201.64 |
| V | GTG | 0.31 | 205.85 | 0.49 | 296.35 |
| V | GTT | 3.79 | 454.43 | 2.42 | 1227.05 |
| Y | TAC | 2.04 | 54.68 | 1.24 | 27.26 |
| Y | TAT | 0.49 | 54.68 | 0.81 | 27.26 |

*Bacillus thuringiensis*

|  |  | **CEM** | | **corseq** | |
| --- | --- | --- | --- | --- | --- |
| **AA** | **Codon** | **Odds ratio** | **Significance (Z^2^-score)** | **Odds ratio** | **Significance (Z^2^-score)** |
| A | GCA | 0.94 | 1.54 | 0.95 | 1.82 |
| A | GCC | 0.61 | 21.06 | 0.53 | 27.06 |
| A | GCG | 0.79 | 16.39 | 0.91 | 2.83 |
| A | GCT | 1.38 | 48.65 | 1.19 | 22.37 |
| C | TGC | 0.93 | 0.20 | 0.72 | 3.41 |
| C | TGT | 1.08 | 0.20 | 1.38 | 3.41 |
| E | GAA | 1.08 | 2.49 | 0.90 | 5.63 |
| E | GAG | 0.93 | 2.49 | 1.11 | 5.63 |
| D | GAC | 1.76 | 82.25 | 1.22 | 16.28 |
| D | GAT | 0.57 | 82.25 | 0.82 | 16.28 |
| G | GGA | 0.75 | 38.64 | 0.95 | 1.59 |
| G | GGC | 1.24 | 10.47 | 0.88 | 8.34 |
| G | GGG | 0.54 | 68.79 | 0.47 | 158.85 |
| G | GGT | 1.55 | 95.84 | 1.46 | 145.78 |
| F | TTC | 1.84 | 115.29 | 1.43 | 29.25 |
| F | TTT | 0.54 | 115.29 | 0.70 | 29.25 |
| I | ATA | 0.54 | 117.54 | 0.37 | 61.37 |
| I | ATC | 2.15 | 232.07 | 1.47 | 78.77 |
| I | ATT | 0.88 | 8.95 | 0.82 | 20.46 |
| H | CAC | 1.55 | 22.01 | 1.26 | 7.96 |
| H | CAT | 0.65 | 22.01 | 0.79 | 7.96 |
| K | AAA | 1.08 | 2.44 | 1.10 | 3.20 |
| K | AAG | 0.93 | 2.44 | 0.91 | 3.20 |
| L | CTA | 1.03 | 0.22 | 0.85 | 5.10 |
| L | CTC | 0.66 | 16.83 | 0.42 | 18.00 |
| L | CTG | 0.70 | 10.25 | 0.56 | 13.20 |
| L | CTT | 1.22 | 18.16 | 1.27 | 29.84 |
| L | TTA | 1.01 | 0.07 | 1.05 | 1.19 |
| L | TTG | 0.89 | 3.16 | 0.60 | 20.47 |
| N | AAC | 2.06 | 163.27 | 1.42 | 33.01 |
| N | AAT | 0.49 | 163.27 | 0.70 | 33.01 |
| Q | CAA | 1.51 | 26.96 | 1.39 | 11.45 |
| Q | CAG | 0.66 | 26.96 | 0.72 | 11.45 |
| P | CCA | 1.15 | 4.42 | 1.14 | 4.55 |
| P | CCC | 0.68 | 3.39 | 0.15 | 35.84 |
| P | CCG | 0.75 | 11.63 | 0.79 | 6.23 |
| P | CCT | 1.11 | 2.01 | 1.18 | 5.85 |
| S | AGC | 1.17 | 4.11 | 1.06 | 0.43 |
| S | AGT | 0.77 | 18.91 | 0.87 | 3.81 |
| S | TCA | 0.89 | 4.27 | 0.88 | 3.58 |
| S | TCC | 0.78 | 4.95 | 0.48 | 12.51 |
| S | TCG | 0.72 | 11.85 | 0.39 | 24.58 |
| S | TCT | 1.53 | 65.63 | 1.38 | 37.49 |
| R | AGA | 0.62 | 45.80 | 0.52 | 41.29 |
| R | AGG | 0.28 | 77.88 | 0.17 | 26.87 |
| R | CGA | 0.45 | 75.97 | 0.46 | 38.03 |
| R | CGC | 1.62 | 33.53 | 1.13 | 3.86 |
| R | CGG | 0.26 | 43.19 | 0.07 | 72.78 |
| R | CGT | 2.42 | 235.05 | 1.75 | 116.14 |
| T | ACA | 0.96 | 0.69 | 0.92 | 2.80 |
| T | ACC | 0.69 | 8.61 | 0.26 | 32.80 |
| T | ACG | 0.74 | 23.61 | 0.74 | 18.97 |
| T | ACT | 1.47 | 49.31 | 1.42 | 54.72 |
| V | GTA | 1.05 | 1.22 | 1.09 | 5.21 |
| V | GTC | 0.61 | 32.44 | 0.41 | 49.36 |
| V | GTG | 0.76 | 19.85 | 0.83 | 7.62 |
| V | GTT | 1.26 | 28.03 | 1.09 | 5.43 |
| Y | TAC | 1.57 | 43.79 | 1.60 | 46.29 |
| Y | TAT | 0.64 | 43.79 | 0.63 | 46.29 |

*Saccharomyces cerevisiae*

|  |  | **CEM** | | **corseq** | |
| --- | --- | --- | --- | --- | --- |
| **AA** | **Codon** | **Odds ratio** | **Significance (Z^2^-score)** | **Odds ratio** | **Significance (Z^2^-score)** |
| A | GCA | 0.10 | 64.33 | 0.18 | 2251.72 |
| A | GCC | 1.74 | 10.35 | 1.02 | 0.86 |
| A | GCG | 0.42 | 6.64 | 0.11 | 927.60 |
| A | GCT | 2.97 | 52.13 | 3.07 | 3347.34 |
| C | TGC | 0.27 | 11.48 | 0.36 | 221.63 |
| C | TGT | 3.74 | 11.48 | 2.81 | 221.63 |
| E | GAA | 3.31 | 21.54 | 5.09 | 1688.03 |
| E | GAG | 0.30 | 21.54 | 0.20 | 1688.03 |
| D | GAC | 3.17 | 33.96 | 2.11 | 1026.54 |
| D | GAT | 0.32 | 33.96 | 0.47 | 1026.54 |
| G | GGA | 0.16 | 30.25 | 0.34 | 758.42 |
| G | GGC | 0.47 | 9.05 | 0.40 | 601.03 |
| G | GGG | 0.10 | 19.11 | 0.16 | 710.07 |
| G | GGT | 7.11 | 109.10 | 4.25 | 3176.73 |
| F | TTC | 2.85 | 21.24 | 3.42 | 1726.07 |
| F | TTT | 0.35 | 21.24 | 0.29 | 1726.07 |
| I | ATA | 0.17 | 34.87 | 0.25 | 1134.48 |
| I | ATC | 3.02 | 36.28 | 2.23 | 1304.69 |
| I | ATT | 1.06 | 0.03 | 0.89 | 27.83 |
| H | CAC | 6.21 | 50.52 | 2.82 | 951.52 |
| H | CAT | 0.16 | 50.52 | 0.35 | 951.52 |
| K | AAA | 0.24 | 91.60 | 0.21 | 4571.74 |
| K | AAG | 4.10 | 91.60 | 4.73 | 4571.74 |
| L | CTA | 0.28 | 13.49 | 0.44 | 473.20 |
| L | CTC | 0.34 | 4.50 | 0.55 | 130.91 |
| L | CTG | 0.60 | 2.93 | 0.24 | 734.75 |
| L | CTT | 0.32 | 11.48 | 0.96 | 1.81 |
| L | TTA | 0.62 | 5.14 | 0.54 | 584.22 |
| L | TTG | 4.92 | 117.77 | 3.15 | 3906.91 |
| N | AAC | 3.17 | 30.90 | 3.56 | 2314.63 |
| N | AAT | 0.32 | 30.90 | 0.28 | 2314.63 |
| Q | CAA | 4.13 | 22.17 | 4.83 | 1136.41 |
| Q | CAG | 0.24 | 22.17 | 0.21 | 1136.41 |
| P | CCA | 3.43 | 37.12 | 3.49 | 2162.58 |
| P | CCC | 0.31 | 8.57 | 0.36 | 417.89 |
| P | CCG | 0.29 | 5.74 | 0.41 | 237.64 |
| P | CCT | 0.67 | 2.28 | 0.44 | 590.90 |
| S | AGC | 0.58 | 2.84 | 0.88 | 15.55 |
| S | AGT | 0.67 | 2.14 | 0.41 | 546.86 |
| S | TCA | 0.39 | 13.21 | 0.45 | 658.04 |
| S | TCC | 2.20 | 20.61 | 1.68 | 579.82 |
| S | TCG | 0.51 | 3.88 | 0.56 | 199.25 |
| S | TCT | 1.97 | 18.19 | 1.81 | 972.49 |
| R | AGA | 2.87 | 39.91 | 2.46 | 1335.39 |
| R | AGG | 0.35 | 15.03 | 0.18 | 1077.76 |
| R | CGA | 0.14 | 11.35 | 0.65 | 50.55 |
| R | CGC | 0.20 | 6.67 | 0.71 | 37.07 |
| R | CGG | 0.17 | 5.17 | 0.53 | 61.83 |
| R | CGT | 1.55 | 4.36 | 0.91 | 8.23 |
| T | ACA | 0.29 | 24.95 | 0.31 | 1462.31 |
| T | ACC | 2.76 | 33.94 | 2.42 | 2013.36 |
| T | ACG | 0.25 | 12.75 | 0.20 | 873.06 |
| T | ACT | 1.69 | 8.58 | 1.34 | 206.44 |
| V | GTA | 0.17 | 32.12 | 0.27 | 1116.88 |
| V | GTC | 2.02 | 15.02 | 2.24 | 1681.24 |
| V | GTG | 0.23 | 23.49 | 0.17 | 1480.75 |
| V | GTT | 2.57 | 34.75 | 1.47 | 389.90 |
| Y | TAC | 6.22 | 41.14 | 4.26 | 1680.97 |
| Y | TAT | 0.16 | 41.14 | 0.23 | 1680.97 |

*Fusarium graminearum*

|  |  | **CEM** | | **corseq** | |
| --- | --- | --- | --- | --- | --- |
| **AA** | **Codon** | **Odds ratio** | **Significance (Z^2^-score)** | **Odds ratio** | **Significance (Z^2^-score)** |
| A | GCA | 0.33 | 952.20 | 0.54 | 55.93 |
| A | GCC | 1.92 | 683.10 | 1.32 | 32.70 |
| A | GCG | 0.51 | 252.99 | 0.82 | 6.28 |
| A | GCT | 1.32 | 122.60 | 1.09 | 2.63 |
| C | TGC | 2.00 | 110.29 | 1.34 | 4.49 |
| C | TGT | 0.50 | 110.29 | 0.75 | 4.49 |
| E | GAA | 0.37 | 944.94 | 0.21 | 293.72 |
| E | GAG | 2.69 | 944.94 | 4.82 | 293.72 |
| D | GAC | 1.69 | 294.86 | 1.89 | 151.82 |
| D | GAT | 0.59 | 294.86 | 0.53 | 151.82 |
| G | GGA | 0.58 | 268.72 | 0.63 | 60.92 |
| G | GGC | 1.42 | 169.24 | 1.61 | 142.10 |
| G | GGG | 0.23 | 543.15 | 0.76 | 16.90 |
| G | GGT | 1.54 | 253.88 | 0.89 | 7.51 |
| F | TTC | 2.15 | 378.52 | 0.92 | 1.90 |
| F | TTT | 0.47 | 378.52 | 1.09 | 1.90 |
| I | ATA | 0.18 | 577.95 | 0.30 | 85.14 |
| I | ATC | 1.99 | 446.87 | 1.97 | 188.46 |
| I | ATT | 0.83 | 28.64 | 0.62 | 85.15 |
| H | CAC | 2.92 | 458.70 | 2.23 | 51.52 |
| H | CAT | 0.34 | 458.70 | 0.45 | 51.52 |
| K | AAA | 0.19 | 1616.59 | 0.19 | 192.91 |
| K | AAG | 5.20 | 1616.59 | 5.23 | 192.91 |
| L | CTA | 0.43 | 253.44 | 0.39 | 57.51 |
| L | CTC | 2.32 | 1048.68 | 1.36 | 61.22 |
| L | CTG | 0.89 | 10.20 | 1.22 | 19.60 |
| L | CTT | 0.97 | 0.81 | 1.00 | 0.00 |
| L | TTA | 0.30 | 227.22 | 0.01 | 78.36 |
| L | TTG | 0.58 | 202.55 | 0.61 | 50.99 |
| N | AAC | 3.29 | 741.50 | 1.36 | 15.52 |
| N | AAT | 0.30 | 741.50 | 0.74 | 15.52 |
| Q | CAA | 0.46 | 427.19 | 0.25 | 212.58 |
| Q | CAG | 2.16 | 427.19 | 3.94 | 212.58 |
| P | CCA | 0.35 | 623.86 | 0.86 | 3.27 |
| P | CCC | 2.37 | 734.28 | 1.24 | 11.41 |
| P | CCG | 0.36 | 303.13 | 1.46 | 26.10 |
| P | CCT | 1.32 | 75.51 | 0.66 | 34.38 |
| S | AGC | 1.30 | 65.75 | 1.16 | 4.52 |
| S | AGT | 0.47 | 266.95 | 0.51 | 26.72 |
| S | TCA | 0.52 | 285.67 | 0.87 | 3.76 |
| S | TCC | 2.05 | 566.62 | 1.31 | 24.64 |
| S | TCG | 0.57 | 161.97 | 0.84 | 5.31 |
| S | TCT | 1.28 | 69.46 | 1.00 | 0.00 |
| R | AGA | 0.35 | 416.08 | 0.52 | 35.44 |
| R | AGG | 0.40 | 227.62 | 2.10 | 166.70 |
| R | CGA | 1.23 | 33.38 | 0.06 | 253.96 |
| R | CGC | 1.96 | 389.89 | 1.19 | 8.47 |
| R | CGG | 0.37 | 195.04 | 0.43 | 45.58 |
| R | CGT | 1.61 | 170.27 | 1.56 | 65.20 |
| T | ACA | 0.48 | 413.50 | 0.38 | 144.77 |
| T | ACC | 2.62 | 1073.40 | 2.36 | 309.26 |
| T | ACG | 0.42 | 307.82 | 0.60 | 30.21 |
| T | ACT | 0.98 | 0.41 | 0.73 | 27.16 |
| V | GTA | 0.30 | 399.60 | 0.30 | 61.11 |
| V | GTC | 2.17 | 747.23 | 0.79 | 25.08 |
| V | GTG | 0.44 | 342.35 | 1.61 | 98.10 |
| V | GTT | 0.97 | 0.95 | 1.01 | 0.01 |
| Y | TAC | 2.82 | 498.82 | 1.22 | 3.51 |
| Y | TAT | 0.35 | 498.82 | 0.82 | 3.51 |

*Mus musculus*

|  |  | **CEM** | | **corseq** | |
| --- | --- | --- | --- | --- | --- |
| **AA** | **Codon** | **Odds ratio** | **Significance (Z^2^-score)** | **Odds ratio** | **Significance (Z^2^-score)** |
| A | GCA | 0.74 | 136.48 | 0.72 | 514.01 |
| A | GCC | 1.20 | 75.95 | 1.24 | 359.80 |
| A | GCG | 1.48 | 137.19 | 0.81 | 100.14 |
| A | GCT | 0.88 | 31.04 | 1.09 | 52.77 |
| C | TGC | 1.24 | 32.68 | 1.32 | 161.69 |
| C | TGT | 0.81 | 32.68 | 0.76 | 161.69 |
| E | GAA | 0.81 | 85.31 | 0.75 | 536.25 |
| E | GAG | 1.23 | 85.31 | 1.33 | 536.25 |
| D | GAC | 1.10 | 15.32 | 1.11 | 57.26 |
| D | GAT | 0.91 | 15.32 | 0.90 | 57.26 |
| G | GGA | 0.82 | 62.18 | 0.81 | 241.05 |
| G | GGC | 1.23 | 86.28 | 1.23 | 316.98 |
| G | GGG | 0.91 | 13.14 | 0.79 | 287.20 |
| G | GGT | 1.05 | 2.82 | 1.22 | 208.40 |
| F | TTC | 1.26 | 62.71 | 1.32 | 315.64 |
| F | TTT | 0.79 | 62.71 | 0.76 | 315.64 |
| I | ATA | 0.62 | 151.35 | 0.53 | 859.03 |
| I | ATC | 1.31 | 107.05 | 1.32 | 481.54 |
| I | ATT | 0.97 | 1.41 | 0.99 | 0.33 |
| H | CAC | 1.26 | 39.48 | 1.11 | 23.08 |
| H | CAT | 0.79 | 39.48 | 0.90 | 23.08 |
| K | AAA | 0.73 | 182.11 | 0.70 | 869.70 |
| K | AAG | 1.37 | 182.11 | 1.42 | 869.70 |
| L | CTA | 0.82 | 32.60 | 0.91 | 25.57 |
| L | CTC | 1.11 | 20.27 | 0.98 | 2.97 |
| L | CTG | 1.27 | 176.57 | 1.31 | 744.03 |
| L | CTT | 0.90 | 14.59 | 0.86 | 94.26 |
| L | TTA | 0.56 | 209.75 | 0.57 | 525.40 |
| L | TTG | 0.88 | 23.43 | 0.91 | 37.63 |
| N | AAC | 1.23 | 48.49 | 1.37 | 426.17 |
| N | AAT | 0.81 | 48.49 | 0.73 | 426.17 |
| Q | CAA | 0.84 | 29.36 | 0.83 | 107.14 |
| Q | CAG | 1.19 | 29.36 | 1.21 | 107.14 |
| P | CCA | 0.77 | 97.03 | 0.88 | 68.93 |
| P | CCC | 1.22 | 64.18 | 1.16 | 108.34 |
| P | CCG | 1.48 | 118.75 | 0.77 | 116.87 |
| P | CCT | 0.88 | 26.11 | 1.08 | 25.24 |
| S | AGC | 1.20 | 58.23 | 1.04 | 8.31 |
| S | AGT | 0.84 | 34.50 | 0.82 | 119.62 |
| S | TCA | 0.69 | 140.12 | 0.75 | 226.89 |
| S | TCC | 1.15 | 30.24 | 1.27 | 302.22 |
| S | TCG | 1.45 | 73.53 | 0.96 | 2.35 |
| S | TCT | 0.94 | 5.46 | 1.05 | 10.55 |
| R | AGA | 0.77 | 76.41 | 0.78 | 210.96 |
| R | AGG | 0.93 | 5.79 | 0.83 | 116.06 |
| R | CGA | 0.95 | 1.83 | 0.95 | 6.44 |
| R | CGC | 1.36 | 95.56 | 1.39 | 421.47 |
| R | CGG | 1.03 | 1.09 | 0.88 | 57.47 |
| R | CGT | 1.19 | 17.01 | 1.55 | 521.66 |
| T | ACA | 0.80 | 69.44 | 0.72 | 489.15 |
| T | ACC | 1.28 | 96.55 | 1.43 | 791.71 |
| T | ACG | 1.10 | 6.29 | 0.78 | 124.90 |
| T | ACT | 0.90 | 13.10 | 1.01 | 0.63 |
| V | GTA | 0.77 | 56.14 | 0.75 | 203.96 |
| V | GTC | 1.11 | 18.18 | 1.05 | 12.15 |
| V | GTG | 1.10 | 20.44 | 1.12 | 94.55 |
| V | GTT | 0.89 | 16.54 | 0.93 | 19.67 |
| Y | TAC | 1.30 | 59.35 | 1.42 | 379.51 |
| Y | TAT | 0.77 | 59.35 | 0.71 | 379.51 |

*Drosophila melanogaster*

|  |  | **CEM** | | **corseq** | |
| --- | --- | --- | --- | --- | --- |
| **AA** | **Codon** | **Odds ratio** | **Significance (Z^2^-score)** | **Odds ratio** | **Significance (Z^2^-score)** |
| A | GCA | 0.55 | 256.06 | 0.38 | 1125.64 |
| A | GCC | 1.61 | 381.93 | 2.09 | 2242.82 |
| A | GCG | 0.69 | 118.67 | 0.35 | 1407.82 |
| A | GCT | 1.05 | 2.31 | 1.14 | 49.83 |
| C | TGC | 1.52 | 45.62 | 2.06 | 207.96 |
| C | TGT | 0.66 | 45.62 | 0.49 | 207.96 |
| E | GAA | 0.64 | 212.82 | 0.44 | 1212.51 |
| E | GAG | 1.55 | 212.82 | 2.27 | 1212.51 |
| D | GAC | 1.33 | 97.52 | 1.29 | 168.87 |
| D | GAT | 0.75 | 97.52 | 0.77 | 168.87 |
| G | GGA | 0.85 | 27.82 | 0.89 | 31.93 |
| G | GGC | 1.28 | 93.19 | 1.04 | 5.28 |
| G | GGG | 0.87 | 8.20 | 0.29 | 509.23 |
| G | GGT | 0.88 | 15.81 | 1.38 | 286.65 |
| F | TTC | 2.33 | 389.24 | 2.94 | 1092.84 |
| F | TTT | 0.43 | 389.24 | 0.34 | 1092.84 |
| I | ATA | 0.42 | 334.35 | 0.22 | 1267.31 |
| I | ATC | 1.92 | 438.72 | 2.35 | 1710.12 |
| I | ATT | 0.79 | 48.53 | 0.71 | 243.76 |
| H | CAC | 1.34 | 43.82 | 1.63 | 191.89 |
| H | CAT | 0.75 | 43.82 | 0.61 | 191.89 |
| K | AAA | 0.43 | 563.59 | 0.24 | 3998.57 |
| K | AAG | 2.31 | 563.59 | 4.19 | 3998.57 |
| L | CTA | 0.55 | 157.45 | 0.24 | 765.42 |
| L | CTC | 1.31 | 83.21 | 0.87 | 36.03 |
| L | CTG | 1.34 | 162.47 | 1.85 | 1397.30 |
| L | CTT | 1.18 | 19.76 | 0.64 | 182.72 |
| L | TTA | 0.31 | 222.83 | 0.41 | 234.27 |
| L | TTG | 0.68 | 137.50 | 0.89 | 24.73 |
| N | AAC | 1.63 | 214.79 | 3.13 | 1662.32 |
| N | AAT | 0.61 | 214.79 | 0.32 | 1662.32 |
| Q | CAA | 0.58 | 200.47 | 0.37 | 944.37 |
| Q | CAG | 1.72 | 200.47 | 2.71 | 944.37 |
| P | CCA | 0.83 | 25.05 | 0.60 | 324.42 |
| P | CCC | 1.50 | 169.36 | 2.44 | 1869.45 |
| P | CCG | 0.84 | 27.27 | 0.49 | 671.98 |
| P | CCT | 0.79 | 22.49 | 0.87 | 17.62 |
| S | AGC | 0.97 | 1.24 | 0.82 | 67.89 |
| S | AGT | 0.55 | 192.71 | 0.29 | 675.74 |
| S | TCA | 0.58 | 103.55 | 0.42 | 325.99 |
| S | TCC | 1.56 | 249.13 | 1.90 | 1036.72 |
| S | TCG | 1.12 | 12.63 | 1.13 | 25.07 |
| S | TCT | 1.03 | 0.41 | 1.14 | 15.74 |
| R | AGA | 0.60 | 82.55 | 0.30 | 537.08 |
| R | AGG | 0.93 | 2.12 | 0.79 | 50.42 |
| R | CGA | 0.67 | 86.07 | 0.29 | 952.63 |
| R | CGC | 1.52 | 199.52 | 1.81 | 1134.45 |
| R | CGG | 0.79 | 30.33 | 0.26 | 1069.94 |
| R | CGT | 1.20 | 23.41 | 2.01 | 1305.05 |
| T | ACA | 0.56 | 195.30 | 0.70 | 218.33 |
| T | ACC | 1.94 | 520.47 | 2.16 | 1882.51 |
| T | ACG | 0.74 | 79.32 | 0.49 | 808.08 |
| T | ACT | 0.82 | 20.53 | 0.79 | 73.51 |
| V | GTA | 0.58 | 116.56 | 0.31 | 650.17 |
| V | GTC | 1.36 | 99.69 | 1.56 | 549.65 |
| V | GTG | 1.01 | 0.09 | 1.05 | 6.33 |
| V | GTT | 0.91 | 7.69 | 0.79 | 91.15 |
| Y | TAC | 1.91 | 203.77 | 2.20 | 556.78 |
| Y | TAT | 0.52 | 203.77 | 0.46 | 556.78 |

*Caenorhabditis elegans*

|  |  | **CEM** | | **corseq** | |
| --- | --- | --- | --- | --- | --- |
| **AA** | **Codon** | **Odds ratio** | **Significance (Z^2^-score)** | **Odds ratio** | **Significance (Z^2^-score)** |
| A | GCA | 0.70 | 131.74 | 0.14 | 1596.11 |
| A | GCC | 1.44 | 132.53 | 2.70 | 1711.06 |
| A | GCG | 0.66 | 94.94 | 0.08 | 747.79 |
| A | GCT | 1.25 | 66.88 | 1.54 | 309.67 |
| C | TGC | 1.47 | 54.90 | 6.98 | 760.56 |
| C | TGT | 0.68 | 54.90 | 0.14 | 760.56 |
| E | GAA | 0.96 | 2.33 | 0.31 | 2772.46 |
| E | GAG | 1.04 | 2.33 | 3.21 | 2772.46 |
| D | GAC | 1.23 | 45.57 | 2.64 | 942.42 |
| D | GAT | 0.81 | 45.57 | 0.38 | 942.42 |
| G | GGA | 1.43 | 146.10 | 3.98 | 1218.24 |
| G | GGC | 0.79 | 27.93 | 0.22 | 354.92 |
| G | GGG | 0.62 | 60.22 | 0.28 | 148.54 |
| G | GGT | 0.82 | 28.77 | 0.36 | 422.07 |
| F | TTC | 1.65 | 193.44 | 5.40 | 1243.53 |
| F | TTT | 0.61 | 193.44 | 0.19 | 1243.53 |
| I | ATA | 0.52 | 160.79 | 0.07 | 483.96 |
| I | ATC | 1.51 | 175.60 | 4.51 | 2948.48 |
| I | ATT | 0.89 | 15.52 | 0.31 | 1660.84 |
| H | CAC | 1.27 | 22.33 | 4.69 | 1027.90 |
| H | CAT | 0.79 | 22.33 | 0.21 | 1027.90 |
| K | AAA | 0.73 | 134.54 | 0.09 | 6312.94 |
| K | AAG | 1.36 | 134.54 | 11.21 | 6312.94 |
| L | CTA | 0.64 | 73.32 | 0.07 | 488.73 |
| L | CTC | 1.50 | 174.41 | 2.67 | 1992.76 |
| L | CTG | 0.80 | 34.37 | 0.13 | 904.36 |
| L | CTT | 1.24 | 55.82 | 1.88 | 794.09 |
| L | TTA | 0.68 | 66.45 | 0.16 | 460.36 |
| L | TTG | 0.89 | 13.04 | 0.41 | 624.73 |
| N | AAC | 1.38 | 90.45 | 7.47 | 3399.88 |
| N | AAT | 0.72 | 90.45 | 0.13 | 3399.88 |
| Q | CAA | 1.07 | 3.66 | 1.82 | 334.85 |
| Q | CAG | 0.93 | 3.66 | 0.55 | 334.85 |
| P | CCA | 1.67 | 229.96 | 14.85 | 2332.20 |
| P | CCC | 0.71 | 26.62 | 0.13 | 253.51 |
| P | CCG | 0.68 | 79.91 | 0.09 | 732.22 |
| P | CCT | 0.74 | 41.57 | 0.08 | 633.26 |
| S | AGC | 1.05 | 1.06 | 1.03 | 0.38 |
| S | AGT | 0.77 | 42.71 | 0.09 | 766.29 |
| S | TCA | 0.84 | 34.00 | 0.30 | 753.82 |
| S | TCC | 1.26 | 40.14 | 4.65 | 3887.46 |
| S | TCG | 1.00 | 0.01 | 0.26 | 569.32 |
| S | TCT | 1.21 | 35.97 | 1.67 | 334.21 |
| R | AGA | 0.89 | 9.83 | 0.74 | 99.81 |
| R | AGG | 0.55 | 68.42 | 0.11 | 246.22 |
| R | CGA | 0.69 | 92.73 | 0.03 | 1671.85 |
| R | CGC | 1.77 | 156.88 | 3.79 | 2866.14 |
| R | CGG | 0.61 | 70.16 | 0.04 | 481.14 |
| R | CGT | 1.66 | 215.26 | 1.89 | 657.74 |
| T | ACA | 0.77 | 66.24 | 0.16 | 1561.92 |
| T | ACC | 1.57 | 145.81 | 5.73 | 5016.96 |
| T | ACG | 0.70 | 66.03 | 0.09 | 799.16 |
| T | ACT | 1.18 | 24.73 | 0.92 | 9.04 |
| V | GTA | 0.70 | 66.29 | 0.20 | 646.46 |
| V | GTC | 1.44 | 125.56 | 2.73 | 1953.16 |
| V | GTG | 0.81 | 34.65 | 0.19 | 1146.84 |
| V | GTT | 1.04 | 1.92 | 1.11 | 20.57 |
| Y | TAC | 1.45 | 77.01 | 8.02 | 2033.63 |
| Y | TAT | 0.69 | 77.01 | 0.12 | 2033.63 |
